# Early Ear, Nose and Throat Manifestations in Eosinophilic Granulomatosis with Poliangioitis: Results from Our Cohort Group and Literature Review

**DOI:** 10.3390/jcm12226967

**Published:** 2023-11-07

**Authors:** Mario D’Onofrio, Daniele La Prova, Maria Rosaria Galdiero, Elena Cantone, Eugenio Tremante, Massimo Mascolo, Vittoria Barbieri, Claudio Di Nola, Giuseppe Spadaro, Amato de Paulis, Aikaterini Detoraki

**Affiliations:** 1 Department of Internal Medicine and Clinical Complexity, Division of Internal Medicine and Clinical Immunology, Azienda Ospedaliera Universitaria Federico II, 80131 Naples, Italy; mariodonofrio94@gmail.com (M.D.); danielelaprovadlp@gmail.com (D.L.P.); mrgaldiero@libero.it (M.R.G.); tarta.ruga1991@hotmail.it (V.B.); depaulis@unina.it (A.d.P.); 2 Center of Basic and Clinical Immunology Research, Centro Interdipartimentale di Ricerca in Scienze Immunologiche di Base e Cliniche (CISI), University of Naples “Federico II”, 80131 Naples, Italy; spadaro@unina.it; 3 Department of Neurosciences, Science of Reproduction and Odontostomatology, Division of ENT, University of Naples “Federico II”, 80131 Naples, Italy; elena.cantone@unina.it (E.C.); claudio.dinola@yahoo.it (C.D.N.); 4 Department of General and Specialistic Surgery, Division of ENT, Azienda Ospedaliera dei Colli, 80131 Naples, Italy; eugeniotremante960@gmail.com; 5 Pathology Section, Department of Advanced Biomedical Sciences, University of Naples “Federico II”, 80131 Naples, Italy; massimo.mascolo@unina.it

**Keywords:** eosinophilic granulomatosis with polyangiitis, EGPA, ENT, rhinosinusitis, CRSwNP, hearing loss, otitis, ANCA, vasculitis

## Abstract

Eosinophilic granulomatosis with polyangiitis (EGPA) is a rare, systemic necrotizing vasculitis affecting small-to-medium-sized vessels. EGPA’s clinical manifestations are heterogeneous, affecting different organs and systems, and the upper respiratory tract can be affected by ear, nose and throat (ENT) involvement. The aim of our study was to assess type manifestations at the time of diagnosis in a cohort of EGPA patients and correlate findings with baseline variables (sex, age, antineutrophil cytoplasmic antibodies—ANCA-status) and literature reports. The main ENT manifestations in our patients at the time of diagnosis were: chronic rhinosinusitis with nasal polyposis (CRSwNP) (52%), turbinate hypertrophy (48%), nasal swelling (40%), rhinorrhea (40%), chronic rhinosinusitis without nasal polyposis (CRSsNP) (32%), nasal bone deformities (32%), nasal crusts (20%), nasal mucosal ulcers (12%), corditis (12%), hoarseness/dysphonia (12%), hearing loss (12%), mucoceles (4%) and eosinophilic rhinitis (4%). No correlations were found between sex, age, ANCA status and ENT clinical manifestations. A polymorphic ENT involvement is often observed in the early stages of EGPA. The presence of nasal, sinus, ear and/or laryngeal manifestations in patients with asthma and hypereosinophilia, independently of sex, age or ANCA status, should raise an alert for further investigation and differential diagnosis for EGPA. ENT specialists should be aware of their leading position in this diagnostic race.

## 1. Introduction

Eosinophilic granulomatosis with polyangiitis (EGPA) is a rare systemic necrotizing vasculitis affecting small-to-medium-sized vessels [1]. EGPA is included in the spectrum of ANCA-associated vasculitis (AAV), as approximately 40% of the patients present antineutrophil cytoplasmic antibodies (ANCA), mainly specific for myeloperoxidase (MPO-ANCA) [2]. Prevalence in the general population is 10.7–13 per million, with an annual incidence estimated at 1–4 per million per year [3]. EGPA’s clinical manifestations are extremely heterogeneous, affecting different organs and systems with variable involvement over time. The disease has unique characteristics as it combines respiratory manifestations with hypereosinophilic disorders and AAV features [4,5]. In EGPA patients, different areas of the upper respiratory tract can be affected by ear, nose and throat (ENT) involvement. The aim of this study was to assess the type of ENT manifestations present at the time of diagnosis in our cohort of EGPA patients and correlate the findings with distinct variables such as sex, age at diagnosis and antineutrophil cytoplasmic antibody (ANCA) status. A correlation of ENT manifestations observed in our EGPA patients with previous reports from the literature was also carried out. 

## 2. Materials and Methods

In this retrospective study conducted by the Division of Internal Medicine and Clinical Immunology of the Azienda Ospedaliera Universitaria Federico II, the medical records of 25 EGPA patients (15 males, 10 females) from the last 10 years (2013–2023) were retrospectively examined to assess the type and prevalence of ENT manifestations during initial hospital access for clinical evaluation and diagnosis. Consultations performed by ear, nose and throat (ENT) hospital specialists in the period immediately preceding the EGPA diagnosis were examined in order to possibly identify initial ENT signs and symptoms of the disease. Inclusion criteria for clinical records research were the final diagnosis of EGPA according to the American College of Rheumatology 1990 criteria [6] and/or the definition of EGPA adopted by the 2012 Chapel Hill Consensus Conference [2] and more recently by the classification criteria of the ACR/EULAR 2022 [7]. Exclusion criteria were the dated diagnosis of EGPA (patients on pharmacological treatment and follow-up at the time of ENT evaluation) and the diagnosis of ANCA-associated vasculitis other than EGPA. For each patient, baseline characteristics (sex, age at diagnosis), laboratory findings (eosinophil count) and ANCA status (MPO-ANCA, PR3-ANCA) at the time of diagnosis were registered (Table 1).

Extra-ENT clinical manifestations (asthma, pulmonary infiltrates, heart involvement, peripheral nervous system involvement, arthralgia/arthritis, ocular involvement, skin involvement, glomerulonephritis) were also assessed. In addition, medical records of computer tomography (CT) or magnetic resonance imaging (MRI) of the maxillary sinus and ENT examination with rhinofibrolaryngoscopy with flexible instruments were reported, together with information on previous surgery for ENT disease. The following ENT features, in accordance with the ENT area of the Birmingham Vasculitis Activity Score (BVAS) [8], were registered:

1. Bloody nasal discharge/crusts/ulcers/granulomata;
2. Paranasal sinus involvement;
3. Subglottic stenosis/corditis/vocal cord paralysis/hoarseness/dysphonia;
4. Conductive hearing loss/tympanic membrane changes/otorrhea.

More in detail, medical records and ENT consultation were also examined for the following features: epistaxis, nasal swelling, rhinorrhea, turbinate hypertrophy, nasal septum perforation, saddle nose, nasal bone deformities, eosinophilic rhinitis, subglottic stenosis, corditis, vocal cord paralysis, hoarseness/dysphonia, hearing loss, tympanic membrane changes, otorrhea and chronic rhinosinusitis with and without nasal polyposis (CRSwNP and CRSsNP, respectively).

All procedures complied with the Helsinski Declaration of 1964, subsequently revised in 2013. Informed consent was obtained from all subjects involved in this study.

### Statistical Analysis

The correlation of baseline features (age, sex and ANCA status) with the different ENT clinical manifestations was tested by Fisher’s exact test analysis. Statistical analysis was performed using Prism 8 (GraphPad Software Ver. 8). Differences were considered statistically significant if the *nb*-value was less than 0.05. The existence of an association between patients’ characteristics and ENT manifestations was also assessed by logistic regression analysis.

## 3. Results

**Figure 1:**
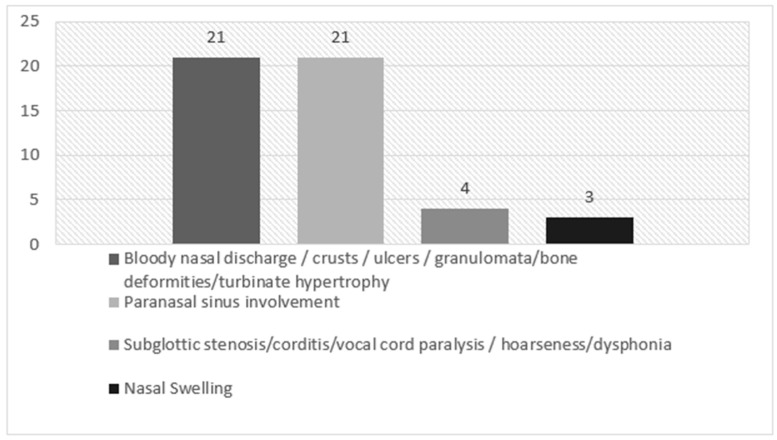
ENT manifestations at the time of diagnosis in our EGPA patients, divided by BVAS area.

In our cohort of EGPA patients, ENT clinical manifestations at the time of diagnosis were chronic rhinosinusitis with nasal polyposis (CRSwNP) (13/25 patients, 52%), turbinate hypertrophy (12/25 patients, 48%), nasal swelling (10/25 patients, 40%), rhinorrhoea (10/25 patients, 40%), chronic rhinosinusitis without nasal polyposis (CRSsNP) (8/25 patients, 32%), nasal bone deformities (8/25 patients, 32%), nasal crusts (5/25 patients, 20%), nasal mucosal ulcers (3/25 patients, 12%), corditis (3/25 patients, 12%), hoarseness/dysphonia (3/25 patients, 12%), hearing loss (3/25 patients, 12%), eosinophilic rhinitis (1/25 patients, 4%) and mucoceles (1/25 patients, 4%). Subglottic stenosis, vocal cord paralysis, tympanic membrane change or otorrhea were not registered at the time of diagnosis. Main ENT manifestations at time of diagnosis in our EGPA patients are reported in Table 2 (divided by BVAS area) and in Figure 1.

**Figure 2:**
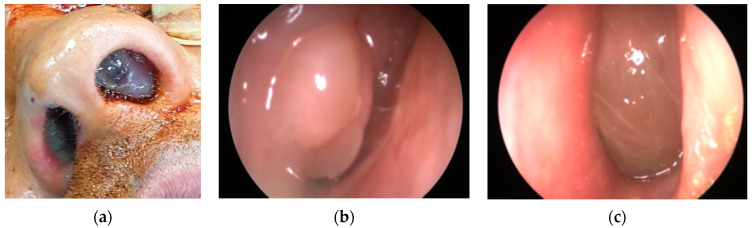
(**a**) Nasal polyposis in a 35-year-old male EGPA patient. (**b**,**c**) Endoscopic image of nasal polyposis in the left and right nostrils.

In our cohort, 13 patients (52%) had CRSwNP. This ENT manifestation was present in our EGPA patients independently of their ANCA status, similarly to previous literature data [9,10]. Figure 2 shows nasal polyps in two of our EGPA patients (Figure 2a–c). In addition, different rhinologic findings were present at the time of diagnosis: turbinate hypertrophy (12 patients, 48%), nasal swelling (10 patients, 40%), rhinorrhoea (10 patients, 40%), CRSsNP (8 patients, 32%), nasal bone deformities (8 patients, 32%), nasal crusts (5 patients, 20%), nasal mucosal ulcers (3 patients, 12%), eosinophilic rhinitis (1 patient, 4%) and mucoceles (1 patient, 4%). Hearing loss was reported in three patients (12%) and was associated with female sex.

**Figure 3:**
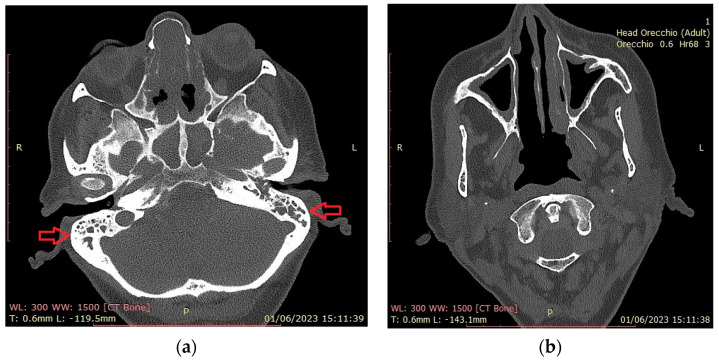
Head CT scan. (**a**) Total obliteration of the mastoid cells (red arrows) by a hyperdense phlogistic tissue. Total obstruction of sphenoidal and subtotal obstruction of etmoid sinuses; (**b**) hypertrophy of the maxillary sinus mucosa. Absence of the right inferior turbinate (asportation by previous surgery).

Figure 3 shows CT findings in an EGPA female patient with mastoiditis, hearing loss and CRSwNP.

Corditis (3 patients, 12%) and hoarseness/dysphonia (3 patients, 12%) were the only laryngeal manifestations in our cohort of patients at the time of diagnosis.

**Figure 4:**
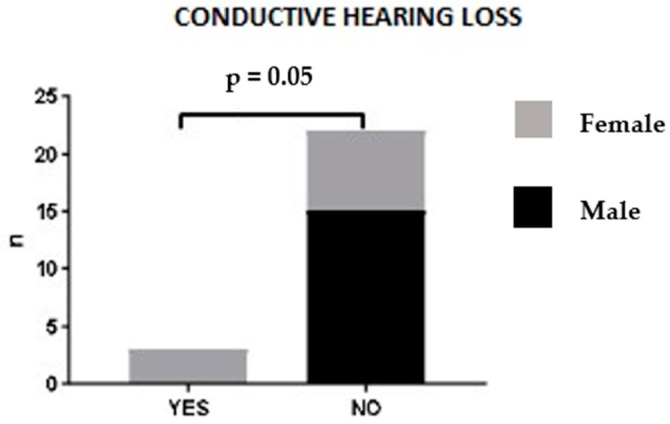
Hearing loss and sex correlation.

No statistically significant correlation was found between sex, age at diagnosis or ANCA status and ENT clinical manifestations. A trend was observed between sex and ENT involvement: in our patient cohort, hearing loss was associated with female sex. Indeed, male patients did not present this ENT manifestation at the time of diagnosis (*p* = 0.052) (Figure 4). Upon logistic regression analysis, no association was found between patients’ clinico-pathological features and ENT manifestations.

**Figure 5:**
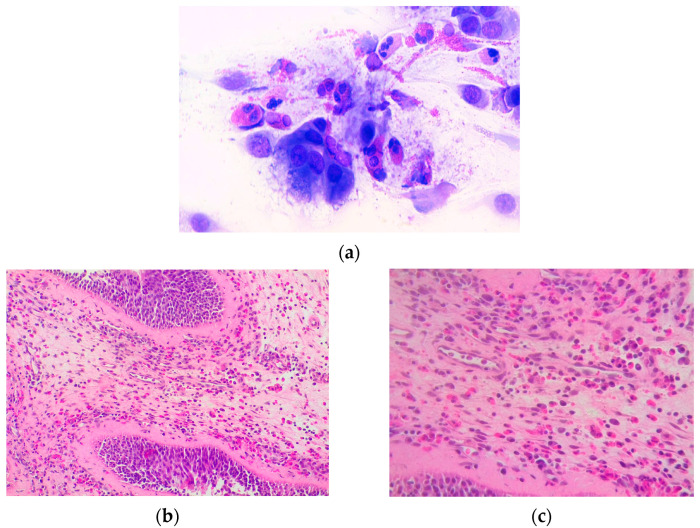
(**a**) Eosinophils with abundant degranulation are observed in the nasal citology findings in a patient with eosinophilic rhinitis (May Grunwald-Giemsa (MGG), 1000×); (**b**,**c**) histological image of nasal polyp with eosinophilic infiltration at various magnifications (20× and 40×).

In our patients, only eosinophilic infiltration in polyp tissue biopsies or nasal cytology was registered (Figure 5a–c).

## 4. Discussion

In the course of EGPA, it is possible to identify a “prodromal” phase characterized by respiratory manifestations of the upper and lower airways, which can even precede systemic manifestations by many years [11]. The second stage of the disease, defined as “eosinophilic” for the presence in circulation of a high quantity of tissue-infiltrating eosinophils, is characterized by systemic involvement with fever, asthenia, weight loss and arthromyalgia, whereas the third phase is defined as “vasculitic”, characterized by the worsening of systemic symptoms and by the involvement of the peripheral nervous, renal and cutaneous systems. ENT manifestations may be present from the very early to the vasculitic stages of the disease, and EGPA diagnosis should always be considered in patients with asthma, sinus involvement and eosinophilia who present systemic involvement (fever, asthenia, weight loss and arthromyalgia) or develop end-organ involvement (lung infiltrates, cardiomyopathy, peripheral neuropathy, glomerulonephritis or other complications) [9].

In our study, the medical records of 25 EGPA patients (15 males, 10 females) from 2013 to 2023 were retrospectively examined to assess the type and prevalence of ENT manifestations during initial hospital access for clinical evaluation and before receiving an EGPA diagnosis. Thus, at the time of evaluation, none of the patients were yet on treatment for EGPA.

The results are represented in a descriptive manner and in decreasing order of prevalence; Table 2, Figure 1. We also divided the results into four ENT areas in accordance with the BVAS questionnaire to highlight aspects that could be considered useful for further assessment during the follow-up phase and treatment.

### 4.1. Nasal and Sinus Involvement

Nasal and sinus involvement is very common and has always been listed among clinical criteria for EGPA diagnosis, since the introduction of 1990′s ACR classification criteria [6] to the 2012 Chapel Hill Consensus Conference [2] and until the publication of the latest ACR/EULAR 2022 classification criteria [7]. Thus, nasal and sinus involvement are central to the ENT manifestations in EGPA and important for its diagnosis. In CRSwNP associated with EGPA, polyps commonly reoccur after surgical removal and frequently coexist with other ENT manifestations, such as middle ear inflammation [12].

A retrospective analysis by Bacciu A et al. on 28 EGPA patients showed evidence of ENT involvement in 21 (75%) at disease onset and/or diagnosis. Only 2/21 (9.5%) received an EGPA diagnosis by an otolaryngologist. The most common ENT manifestations were allergic rhinitis in nine patients (42.8%) and nasal polyposis in 16 patients (76.1%). Three patients (14.2%) developed chronic rhinosinusitis without polyps, and three (14.2%) had nasal crusting [13].

In the first systematic review of the literature on 1175 patients with EGPA, among clinical and cohort study results, 48.0% to 96.0% of patients presented with various rhinologic conditions, including sinusitis, nasal polyps, rhinitis, nasal crusting and nasal obstruction. Patients with documented nasal or paranasal sinus biopsies demonstrated eosinophilic infiltration in 35% to 100% of samples with no evidence of necrotizing vasculitis or eosinophilic granuloma [14].

In a 2018 retrospective observational study, the diagnosis of EGPA was suspected by linking refractory otitis media with effusion (OME) and chronic rhinosinusitis with nasal polyposis, which affected all patients in the study. Almost 60 percent of patients who required grommet insertion for otitis media with effusion (OME) in the setting of nasal polyps met the diagnostic criteria or had a formal diagnosis of EGPA. Thus, the co-existing presence of nasal polyps and resistant OME should raise the possibility of EGPA. The pathophysiology of EGPA indicates that initially rhinological manifestations are the main symptoms, and recognition of nasal polyposis and OME together may lead to earlier referral and subsequent diagnosis of the disease given that the pulmonary manifestations are frequently quiescent [15].

In our cohort, the prevalent ENT manifestation was CRSwNP in 13 patients (52%). Similar to the published data, CRSwNP was not associated with ANCA status [9,10]. Most of these patients (13) had already undergone 1–4 surgical excisions for CRSwNP (Figure 3). Similar to other studies (Brescia et al.) [16], eosinophils were the most abundant cell population in the nasal polyp tissue of our EGPA patients (Figure 5b,c). Interestingly, there was no correlation between eosinophil infiltration, corticosteroid therapy, or peripheral blood eosinophilia. This presumably explains the recalcitrant and recurrent nature of polyposis in EGPA patients [17]. Other rhinologic findings at the time of diagnosis were turbinate hypertrophy (48%), nasal swelling (40%), rhinorrhoea (40%), CRSsNP (32%), nasal bone deformities (32%), nasal crusts (20%), nasal mucosal ulcers (12%), eosinophilic rhinitis (4%) and paranasal sinus mucoceles (4%). The latter are epithelium-lined cystic masses, mucus-filled, resulting from obstruction of the sinus ostia. They are generally benign, characterized by slow growth, but may become potentially harmful to adjacent structures through mass effect [18]. They often occur after surgery for recalcitrant nasal polyposis [19] and overcomplicate CRSwNP management in EGPA [20].

At the time of evaluation, two patients were on treatment with an anti-IL-4/antiIL-13 monoclonal antibody for severe, recurrent CRSwNP, and five patients were on treatment with anti-IL5/anti-IL5Ra therapy for severe eosinophilic asthma. The effect of the above treatments and/or oral steroid therapy prescribed for severe ENT manifestations was not assessed in terms of EGPA diagnosis and/or prognosis in our patients.

### 4.2. Ear Involvement

As for otologic manifestations, serous otitis media, purulent otitis media, unilateral facial palsy and progressive sensorineural hearing loss were reported in a small number of patients (4.7%, 4,7%, 4,7% and 9.5%, respectively) in the retrospective analysis by Bacciu, A. et al. [13]. Otitis media, sensorineural hearing loss, mastoiditis and facial nerve palsies have been reported by the Goldfarb, J. M., et al. systematic review [14]. In addition, in the 2018 Irish Study, sixteen patients were affected by otitis media with effusion (OME) before receiving an EGPA diagnosis [15]. In a more recent systematic review on the diagnosis, treatment and management of patients with otologic manifestations of EGPA, a systematic search for relevant published literature in the PubMed, Cochrane Library and EMBASE databases was performed. Hearing loss and middle ear effusion were commonly observed in EGPA patients [21]. Conductive hearing loss may be secondary to effusion or obstruction caused by eosinophilic granuloma, while middle ear effusion occurs with thick, mucoid aural discharge enriched by a large number of eosinophils [12]. In a recent Korean case-control study, the most prevalent manifestations associated with OME were conductive hearing loss and mixed-type hearing loss. Therefore, OME may cause mild-to-moderate conductive hearing loss. Steroid treatment adequately dosed may prevent ear symptoms and progressive hearing loss in EGPA patients [22]. In addition, neurological involvement most commonly manifests as mononeuritis multiplex or hearing loss in EGPA patients [23]. In the Nakamaru et al. study, more than half of the EGPA patients had mild to moderately severe hearing loss. The most common pattern of hearing impairment was mixed hearing loss, followed by sensorineural hearing loss. The etiology of hearing loss is not clear; it may be a complication of chronic otitis media, although vasculitic mechanisms could be potentially involved given the rapid response to high-dose corticosteroids [24].

In our cohort, hearing loss was reported in three female patients (12%) with concomitant CRSwNP. Figure 3 shows CT findings in an EGPA female patient with mastoiditis, hearing loss and CRSwNP. At the time of the EGPA diagnosis, the patient had already received 6 months of treatment with biological agents (anti IL-4/anti-IL13 abs) for severe recalcitrant CRSwNP. Prednisone therapy started for severe CRswNP slowly reversed hearing impairment in this patient, suggesting a vasculitic involvement.

### 4.3. Laryngeal Involvement

Larynx is also involved in EGPA. In a 1997 case report on a 59-year-old EGPA patient, a video-laryngostroboscopy revealed paresis and hypotonia of the right vocal cord with a reduction in the adduction phase and an apparently normal laryngeal mucosa. The authors reported manifestations likely secondary to a vasculitic process of the vasa nervorum of the vagus nerve, mostly of the superior laryngeal nerve. Another clue supporting this theory was the efficacy of steroid treatment in improving laryngeal symptoms [25]. In a 2005 retrospective analysis, 21 EGPA patients selected from the Secondary and Primary Vasculitides (SE.PRI.VA.) study group of the University of Parma did not report laryngeal involvement [13]. In a more recent study, recurrent laryngeal polyposis was described, and extravascular eosinophilic histology was demonstrated for laryngeal polyps [14]. In another study on 43 EGPA patients, laryngoscopic findings showed no evidence of impairment of laryngeal movements or any specific lesions under conventional white light or narrow-band imaging. Laryngeal hyperaemia was observed in most patients (n = 31; 72%) involving the arytenoids (n = 23, 53.4%), whereas the whole larynx was affected in a few patients (n = 8, 18.6%). Thick endolaryngeal secretions (n = 10, 23.2%) and mild to moderate laryngeal oedema (n = 17, 39.5%) were present. A slight vocal cord swelling was also observed in some patients (n = 11; 25.6%). The above-mentioned features were also confirmed by narrow-band imaging endoscopy, showing microvessel enhancement and dilation. A low mean reflux finding score (3.3 ± 3.2) was registered in most patients, with only seven patients (16.2 %) scoring 7 or higher.

Laryngeal findings in our cohort were corditis (12%) and hoarseness/dysphonia (12%).

While primary laryngeal involvement in EGPA patients is very uncommon, according to the reviews, inflammation is frequently observed as laryngitis or gastroesophageal reflux. Thus, the clinical meaning of laryngeal manifestations in assessing disease activity should probably be reconsidered, as laryngeal manifestations actually count in the BVAS questionnaire only for the item “subglottic stenosis”. Undoubtedly, laryngoscopic examination is necessary for subglottic inflammation and/or stenosis diagnosis [26].

### 4.4. Microscopic Features of Head and Neck Manifestations in EGPA

The examination of tissue samples from ENT regions rarely shows necrotizing vasculitis or eosinophilic granulomas [13]. When present, granulomatous findings in the upper respiratory tract show nuclear fragments of granulocytes in a central necrotic area ringed by a palisade of epithelioid cells and numerous eosinophils. Granulomas and necrotic areas may be confluent, presenting a ‘geographic’ image at low magnification. Biopsy samples from lung or upper airways and cytology specimens from bronchoalveolar lavage or nasal swabs mostly show multinucleated giant cells; these aspects are considered pathognomonic for EGPA if they match the clinical features of AAVs [27]. In the Goldfarb, J.M., et al. systematic review, 48.0% to 96.0% of patients with some head and neck involvement presented eosinophilic infiltration in 35% to 100% of samples from nasal or paranasal sinus biopsies [14].

As previously reported, granulomatosous findings were not observed in ENT biopsy specimens in our patients.

EGPA is a rare vasculitis, and multicenter studies should be considered to generate more significant clinical observations. In this context, our study has some limitations, as the retrospective exploring design and the small number of subjects make it difficult to extend the results to EGPA patients overall. Nevertheless, our results seem not to differentiate this study from existing research. To our knowledge, previous studies on ENT manifestations in EGPA [10,12,13,14,15,21,22,25,26] describe ENT signs and symptoms after diagnosis and/or treatment, showing that different ENT manifestations are commonly present in EGPA but are not pathognomonic. We suggest that these manifestations have been present since the first stages of the disease and before EGPA diagnosis.

## 5. Conclusions

EGPA’s complex pattern of clinical signs and symptoms, laboratory values and histological findings request a multidisciplinary collaboration between allergistsimmunologists, otolaryngologists, pulmonologists, pathologists and other specialists in order to quickly build a valid diagnostic work-up. EGPA is a rare vasculitis, though some of its most frequent ENT findings (CRSwNP, CRSsNP, otitis, etc.) may be commonly diagnosed in the general population. ENT manifestations in EGPA may be polymorphic and precocious, and EGPA diagnosis should always be considered in patients with ENT involvement with comorbid asthma and eosinophilia who develop other clinical complications. ENT specialists should be aware of their leading position in this diagnostic race.

## Figures and Tables

**Table 1:** Baseline characteristics of patients at the time of EGPA diagnosis.

Patients with	No.
**Sex, no**	25
Male	15
Female	10
Mean Age (yrs)	52.08
Age at diagnosis (yrs)	44.75
**Other organ involvement**	
Asthma	25
Heart involvement	11
Pulmonary infiltrates	10
Peripheral Nervous System	8
Skin involvement	8
Arthritis/joint pain	6
Eye involvement	4
Glomerulonephritis	2

**Table 2:** (**a**) Main ENT manifestations at the time of diagnosis in our EGPA patients. (**b**) ENT manifestations at the time of diagnosis in our EGPA patients are divided by BVAS area. No (%).

(a)	
CRSwNP	13 (52%)
Turbinate Hypertrophy	12 (48%)
Nasal Swelling	10 (40%)
Rhinorrhea	10 (40%)
CRSsNP	8 (32%)
Nasal bone deformities	8 (32%)
Nasal Crusts	5 (20%)
Nasal Mucosal Ulcerations	3 (12%)
Corditis	3 (12%)
Hoarseness/Dysphonia	3 (12%)
Hearing Loss	3 (12%)
Eosinophilic Rhinitis	1 (4%)
Mucoceles	1 (4%)
Subglottic Stenosis	0
Vocal Cord Paralysis	0
Tympanic Membrane Changes	0
Otorrhea	0
(**b**)	
Bloody nasal discharge/crusts/ulcers/granulomata/bone deformities/turbinate hypertrophy	21 (84%)
Paranasal sinus involvement	21 (84%)
Subglottic stenosis/corditis/vocal cord paralysis/hoarseness/dysphonia	4 (16%)
Conductive hearing loss/Tympanic membrane changes/Otorrhea	3 (12%)

## Data Availability

Upon demand.
